# Net global warming potential and greenhouse gas intensity as affected by different water management strategies in Chinese double rice-cropping systems

**DOI:** 10.1038/s41598-017-19110-2

**Published:** 2018-01-15

**Authors:** Xiaohong Wu, Wei Wang, Xiaoli Xie, Chunmei Yin, Haijun Hou, Wende Yan, Guangjun Wang

**Affiliations:** 1 Key Laboratory of Agro-ecological Processes in Subtropical Region, Institute of Subtropical Agriculture, Chinese Academy of Sciences, Changsha, 410125 Hunan China; 2 Faculty of Life Science and Technology, Central South University of Forestry and Technology, Changsha, 410004 Hunan China

## Abstract

This study provides a complete account of global warming potential (GWP) and greenhouse gas intensity (GHGI) in relation to a long-term water management experiment in Chinese double-rice cropping systems. The three strategies of water management comprised continuous (year-round) flooding (CF), flooding during the rice season but with drainage during the midseason and harvest time (F-D-F), and irrigation only for flooding during transplanting and the tillering stage (F-RF). The CH_4_ and N_2_O fluxes were measured with the static chamber method. Soil organic carbon (SOC) sequestration rates were estimated based on the changes in the carbon stocks during 1998–2014. Longer periods of soil flooding led to increased CH_4_ emissions, reduced N_2_O emissions, and enhanced SOC sequestration. The net GWPs were 22,497, 8,895, and 1,646 kg CO_2_-equivalent ha^−1^ yr^−1^ for the CF, F-D-F, and F-RF, respectively. The annual rice grain yields were comparable between the F-D-F and CF, but were reduced significantly (by 13%) in the F-RF. The GHGIs were 2.07, 0.87, and 0.18 kg CO_2_-equivalent kg^−1^ grain yr^−1^ for the CF, F-D-F, and F-RF, respectively. These results suggest that F-D-F could be used to maintain the grain yields and simultaneously mitigate the climatic impact of double rice-cropping systems.

## Introduction

By 2015, atmospheric concentrations of CO_2_, CH_4_, and N_2_O had risen steadily to 400 ppm, 1,845 ppb, and 328 ppb, respectively, with an average absolute increase of 2.08 ppm yr^−1^, 6.0 ppb yr^−1^, and 0.89 ppb yr^−1^ over the last 10 years, respectively^1^. Agricultural soils are an important source of CH_4_ and N_2_O, accounting for approximately 60% and 50% of global anthropogenic N_2_O and CH_4_ emissions, respectively, in 2005^2^.

Paddy soils account for the largest area of anthropogenic wetlands in the world and are among the most important sources of greenhouse gases (GHGs). Rice harvest areas in China occupy about 30 million ha, and the hilly areas in southern China play a vital role in rice production of the country^3^. However, considerable differences can exist in the water conditions of the rice fields. Lowland paddies collect abundant runoff and interflow water, and can be flooded almost every day, whereas paddies at higher elevations receive less water. The representative water management regimes practiced currently in the hilly areas in China include continuous flooding (CF), flooding during the rice season except for drainage at midseason and harvest time (F-D-F), and flooding for transplanting and tillering and rain-fed for the rest of the time (F-RF). The midseason drainage has been widely adopted since 1980s. However, in order to reduce water loss, some farmers prefer keeping field flooded rather than applying the midseason drainage. Besides, rice is also traditionally grown under rain-fed condition, mainly due to the lack of irrigation.

Sustainable agriculture requires the minimization of environmental costs. The net global warming potential (GWP) caused by emissions of GHGs could be reduced potentially by the sequestration of soil organic carbon (SOC) and/or the mitigation of CH_4_ and N_2_O emissions. However, agricultural GHGs fluxes are complex and involve many interrelated trade-offs. Generally, SOC sequestration is associated with retarded decomposition of organic materials under anaerobic conditions^4^. CH_4_ is produced from the biological decomposition of organic materials in anaerobic soil environments by methanogens, such as rice grown under flooded conditions^5,6^. N_2_O is generated primarily by the microbial processes of nitrification and denitrification which prefer aerobic soil conditions, such as those created by repeated flooding and drying of paddy soils^5,7–9^. In addition, CH_4_ and N_2_O emissions are enhanced when SOC is improved^10–12^.

There has been an increasing amount of studies on the impact of water management strategies on CH_4_ and N_2_O emissions from paddy fields, but their impact on net GWP has not been studied in detail. Because of the lack of simultaneous measurements of CH_4_ and N_2_O emissions and SOC sequestration over annual rotation cycles, little is known about the annual net GWP from double rice-cropping systems under different water management strategies. The fallow season can be as long as six months in the double rice-cropping systems adopted in southern China; however, the GHGs emissions during the non-rice growth season have not been well documented. In addition, SOC sequestration rates could not be estimated from short-term field experiments. To achieve the dual goals of sustaining productivity and minimizing the negative climatic effects associated with rice cultivation, greenhouse gas intensity (GHGI) has been proposed to integrate climate change concerns with global food production using yield-scaled approach. This is calculated by dividing the GWP by the crop yield, instead of assessing the GHG emissions on an areal basis^13–15^. To the best of our knowledge, little is known about the effect of water management strategies on GHGI in double rice-cropping systems.

This study was conducted on a long-term water management experiment in southern China, initiated in 1998. Field measurements of CH_4_ and N_2_O emissions from double rice-cropping systems under various long-term water management regimes were obtained over one annual rotation cycle of fallow–rice–rice from November 2014 to October 2015. The net ecosystem carbon balance was estimated based on the change in carbon stocks in soil 0–40 cm deep during 1998–2014, and the net GWP and GHGI under different water management strategies were evaluated. The objectives of this study were to expound the effects on GWP and GHGI of the long-term water management strategies adopted in the double rice-cropping systems in southern China, and, thereby, to optimize water management strategies to maintain grain yields and simultaneously mitigate climatic impacts, and to evaluate accurately the climatic effects of rice cultivation.

## Results

### Soil organic carbon sequestration

The long-term water management strategies produced profound effects on the SOC content and soil bulk density. Over a 16-year period (1998–2014), all treatments produced a significant increase in the SOC content (Table 1). Throughout the period 1998–2014, the SOC content in the 0–20 cm soil layer increased at an average rate of 0.240, 0.334, and 0.377 g kg^−1^ yr^−1^ for the F-RF, F-D-F, and CF plots, respectively. And the SOC content in the 20–40 cm soil layer increased at an average rate of 0.053, 0.143, and 0.208 g kg^−1^ yr^−1^ for the F-RF, F-D-F, and CF plots, respectively (Table 1).

**Table 1:** Soil organic carbon sequestration in the 0–20 cm and 20–40 cm soil layers under different water management strategies over the period 1998–2014.

Soil layers	Treatments	γ_1998_ (g cm^−3^)	γ_2014_ (g cm^−3^)	C_1998_ (g kg^−1^)	C_2014_ (g kg^−1^)	ΔCS (kg C ha^−1^)	SOCSR (kg C ha^−1^ yr^−1^)
0–20 cm	F-RF	1.02 ± 0.01 a	0.99 ± 0.01 a	13.12 ± 0.44 a	16.96 ± 0.09 b	8013 ± 998 b	501 ± 62 b
	F-D-F	1.04 ± 0.01 a	0.96 ± 0.02 a,b	12.47 ± 0.22 a	17.81 ± 0.13 a	11074 ± 470 a	692 ± 29 a
	CF	1.04 ± 0.01 a	0.90 ± 0.02 b	12.03 ± 0.23 a	18.07 ± 0.17 a	11854 ± 705 a	741 ± 44 a
20–40 cm	F-RF	1.34 ± 0.02 a	1.33 ± 0.01 a	8.78 ± 0.35 a	9.62 ± 0.37 c	2259 ± 207 c	141 ± 13 c
	F-D-F	1.33 ± 0.01 a	1.26 ± 0.01 b	8.43 ± 0.10 a	10.71 ± 0.20 b	6077 ± 245 b	380 ± 15 b
	CF	1.33 ± 0.01 a	1.19 ± 0.01 c	8.50 ± 0.19 a	11.82 ± 0.25 a	8816 ± 636 a	551 ± 39 a

Mean ± SE. The different letters following each value in the same column indicate significant differences among the treatments in each soil layer (P < 0.05). γ_1998_ and γ_2014_ refer to soil bulk densities, and C_1998_ and C_2014_ refer to the SOC contents in 1998 and 2014, respectively. ΔCS refers to the changes in carbon stocks, and SOCSR refers to the SOC sequestering rate from 1998 to 2014. CF means continuous year-round flooding with 2–10 cm water layer, F-D-F means flooding in rice season, except drainage at midseason and harvest time, and F-RF means flooding for transplanting and tillering, with no further irrigation.

Because the soil bulk density changed from 1998 to 2014 (Table 1), we calculated the SOC stock on an “identical soil mass” basis. Based on the SOC content and bulk density, we estimated that the SOC stock increased by 8,013–11,854 kg C ha^−1^ in the 0–20 cm and by 2,259–8,816 kg C ha^−1^ in 20–40 cm soil layers over the 16-year period of 1998–2014 (Table 1). Correspondingly, annual SOCSRs were estimated at 501–741 and 141–551 kg C ha^−1^ yr^−1^ in the 0–20 cm and 20–40 cm soil layers, respectively. Annual SOCSRs in the 0–40 cm soil layer were 642, 1,072, and 1,292 kg C ha^−1^ yr^−1^ for the F-RF, F-D-F, and CF plots, respectively, with an average of 1,002 kg C ha^−1^ yr^−1^ for the experimental double rice-cropping paddy plots over the period 1998–2014 (Table 1). Longer flooding period not only increased the SOC in the 0–20 cm soil layer, but also promoted the SOC migration into the deeper soil layers. For example, the SOCSR of the F-RF plot in the 20–40 cm soil layer was 28% of that in the 0–20 cm soil layer, whereas the SOCSRs of the F-D-F and CF plots in the 20–40 cm soil layer reached 55% and 74% of those in the 0–20 cm soil layer, respectively.

### CH_4_ emissions

**Figure 1:**
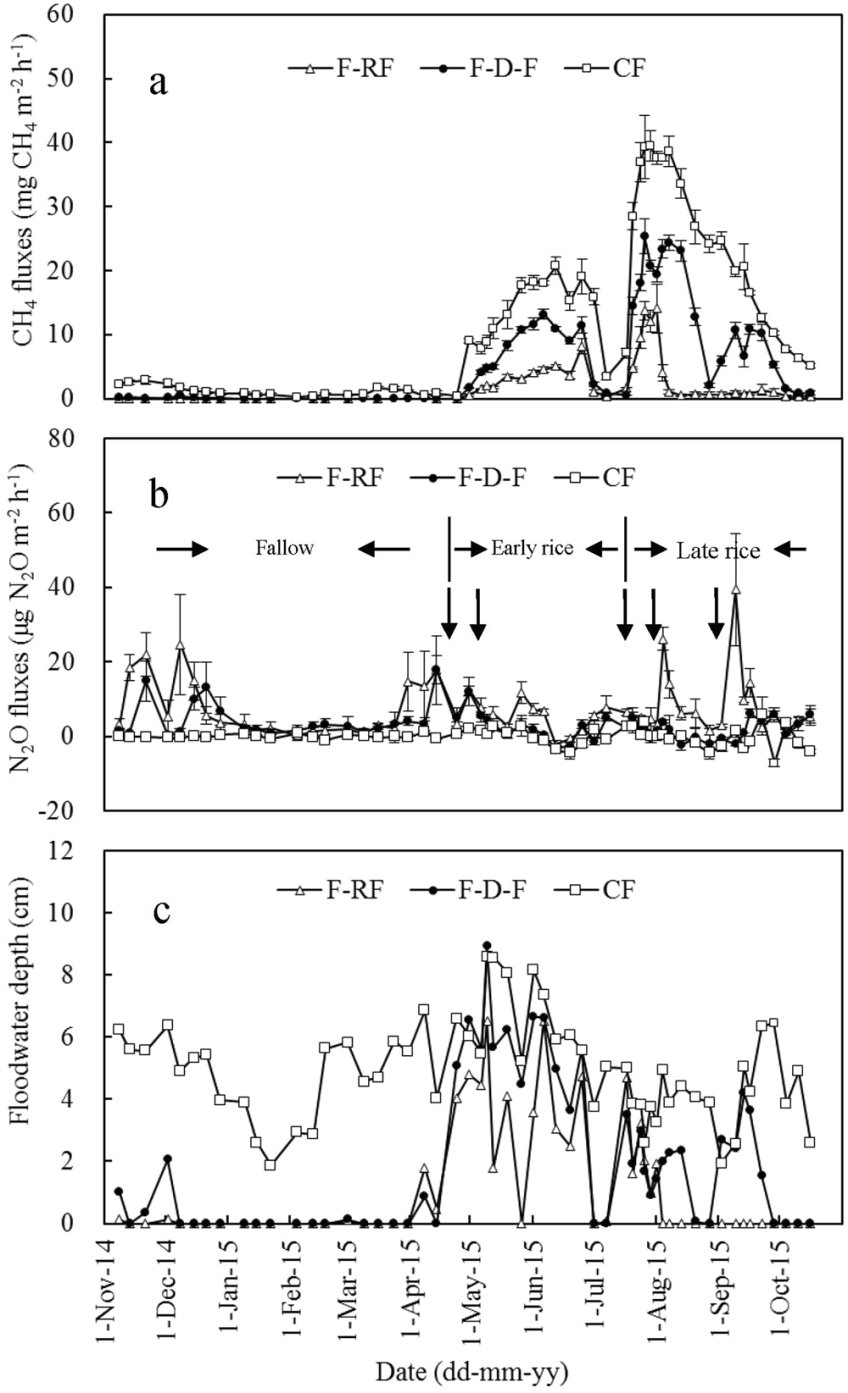
Seasonal variation of CH_4_ (**a**) and N_2_O (**b**) emissions and floodwater depth (**c**) under different water management strategies over an entire annual cycle, from the 2014 fallow season to the 2015 late rice season. The bar with each point indicates the range of the standard error (SE) of the mean. Downward arrows indicate the time of fertilization. See descriptions of CF, F-D-F, and F-RF from Table 1.

The CH_4_ emissions varied significantly with differing season and treatment (Fig. 1a). In the fallow season, the F-RF and F-D-F soils acted as small sources of CH_4_ to the atmosphere, whereas the CF soils acted as a more significant source of CH_4_ (Fig. 1a). The rates of the CH_4_ emissions during the fallow season were extremely low, ranging from 0–1.69 mg m^−2^ h^−1^, at an average rate of 0.01, 0.06, and 1.17 mg CH_4_ m^−2^ h^−1^ for the F-RF, F-D-F, and CF plots, respectively. The cumulative CH_4_ fluxes in the fallow season were 0.5 ± 0.1, 2.5 ± 0.2, and 50.2 ± 6.8 kg CH_4_ ha^−1^ for the F-RF, F-D-F, and CF plots, respectively (Table 2). During the early rice season, the CH_4_ fluxes increased gradually during the early and middle growing stages, but subsequently decreased during the late stage (Fig. 1a). The rates of the CH_4_ emissions during the early rice season ranged from 0.01–20.77 mg CH_4_ m^−2^ h^−1^, at an average rate of 2.91, 7.11, and 13.49 mg CH_4_ m^−2^ h^−1^ for the F-RF, F-D-F, and CF plots, respectively. The cumulative CH_4_ fluxes during the early rice season were 55.2 ± 5.4, 131.4 ± 5.7, and 249.3 ± 14.3 kg CH_4_ ha^−1^ for the F-RF, F-D-F, and CF plots, respectively (Table 2). The CH_4_ fluxes increased dramatically after the transplanting of late rice in mid-July, and reached their peak emissions approximately 10 days after transplanting (Fig. 1a). Subsequently, the CH_4_ fluxes from the CF plots decreased gradually. However, a particularly remarkable reduction of CH_4_ fluxes was noted during the midseason drainage of the F-D-F plots (Fig. 1a). Mostly, when the field was unflooded, the CH_4_ fluxes from the F-RF plots were observed to decrease to approximately 1 mg m^−2^ h^−1^. The rates of the CH_4_ emissions during the late rice season ranged from 0.33–39.46 mg CH_4_ m^−2^ h^−1^, at an average rate of 2.56, 10.97, and 22.89 mg CH_4_ m^−2^ h^−1^ for the F-RF, F-D-F, and CF plots, respectively. The seasonal cumulative CH_4_ fluxes were 56.5 ± 13.5, 242.1 ± 9.8, and 505.3 ± 18.8 kg CH_4_ ha^−1^ for the F-RF, F-D-F, and CF plots, respectively (Table 2). Substantial CH_4_ emissions were observed in the F-D-F and CF plots during the late rice-growing season, i.e., 84% and 103% greater than those during the early rice season. Under the F-RF water management, cumulative CH_4_ fluxes during the late and the early rice seasons were comparable because of the relatively dry conditions during the late rice season. Overall, the annual CH_4_ emissions ranged from 112 ± 19 kg CH_4_ ha^−1^ for the F-RF plots to 805 ± 16 kg CH_4_ ha^−1^ for the CF plots (Table 3). Compared with the F-D-F plots, the annual CH_4_ emissions increased by 114% for the CF plots and decreased by 70% for the F-RF plots.

**Table 2:** Seasonal cumulative CH_4_ and N_2_O emissions and rice grain yield under different water management strategies during the experimental period 2014–2015.

Treatments	Early rice season			Late rice season			Fallow season	
	CH_4_ (kg CH_4_ ha^−1^)	N_2_O (kg N_2_O ha^−1^)	Yield (kg ha^−1^)	CH_4_ (kg CH_4_ ha^−1^)	N_2_O (kg N_2_O ha^−1^)	Yield (kg ha^−1^)	CH_4_ (kg CH_4_ ha^−1^)	N_2_O (kg N_2_O ha^−1^)
F-RF	55.2 ± 5.4 c	0.12 ± 0.02 a	3850 ± 83 b	56.5 ± 13.5 c	0.20 ± 0.03 a	5101 ± 247 b	0.5 ± 0.1 c	0.31 ± 0.09 a
F-D-F	131.4 ± 5.7 b	0.07 ± 0.01 b	3964 ± 99 b	242.1 ± 9.8 b	0.04 ± 0.02 b	6280 ± 179 a	2.5 ± 0.2 b	0.18 ± 0.03 a,b
CF	249.3 ± 14.3 a	0.00 ± 0.01 c	4636 ± 135 a	505.3 ± 18.8 a	−0.01 ± 0.00 c	6250 ± 186 a	50.2 ± 6.8 a	0.00 ± 0.01 b

Mean ± SE. The different letters following each value in the same column indicate significant differences among treatments (P < 0.05). See descriptions of CF, F-D-F, and F-RF from Table 1.

**Table 3:** CH_4_ and N_2_O emissions and soil organic carbon sequestration rates (SOCSR), their estimated global warming potentials (GWP), and greenhouse gas intensities (GHGI) under different water management strategies.

Treatments	CH_4_ kg CH_4_ ha^−1^ yr^−1^	N_2_O kg N_2_O ha^−1^ yr^−1^	SOCSR kg C ha^−1^ yr^−1^	Grain yield kg ha^−1^ yr^−1^	GWP kg CO_2_-eqv ha^−1^ yr^−1^	GHGI kg CO_2_-eqv kg^−1^ grain
F-RF	112 ± 19 c	0.62 ± 0.09 a	642 ± 66 c	8951 ± 173 b	1646 ± 425 c	0.18 ± 0.04 c
F-D-F	376 ± 15 b	0.29 ± 0.05 b	1072 ± 18 b	10244 ± 233 a	8895 ± 513 b	0.87 ± 0.07 b
CF	805 ± 16 a	−0.01 ± 0.00 c	1292 ± 64 a	10886 ± 283 a	22497 ± 593 a	2.07 ± 0.11 a

Mean ± SE. Different letters following each value in the same column indicate significant differences among treatments (P < 0.05). CH_4_ and N_2_O emissions were investigated over an entire annual cycle, from the 2014 fallow season to the 2015 late rice season. SOCSR was estimated from 1998 to 2014. See descriptions of CF, F-D-F, and F-RF from Table 1.

### N_2_O emissions

N_2_O emissions were negligible when the soils were flooded. The CF soils acted as extremely small sinks or sources throughout the annual cycle and there were no obvious N_2_O emissions, even after the application of N fertilizer (Fig. 1b). Relative to the CF soil, the F-D-F and F-RF soils were not flooded usually during the fallow season (Fig. 1c). Larger emissions of N_2_O were observed in the F-D-F and F-RF plots during the fallow season, although no fertilizer was applied (Fig. 1b). The average rates of the N_2_O emissions during the fallow season were 7.1, 4.2, and 0.0 μg N_2_O m^−2^ h^−1^ for the F-RF, F-D-F, and CF plots, respectively. The cumulative N_2_O fluxes during the fallow season were 0.31 ± 0.09, 0.18 ± 0.03, and 0.00 ± 0.01 kg N_2_O ha^−1^ for the F-RF, F-D-F, and CF plots, respectively (Table 2). During the early rice season, there was abundant rainfall that prevented midseason drainage in the F-D-F plots (Fig. 1c). Consequently, the F-D-F soils were flooded until near the end of the early rice season (Fig. 1c). Relative to the F-D-F plots, the water layer in the F-RF plots disappeared briefly in the middle of the growing stage (Fig. 1c). The cumulative N_2_O fluxes in the early rice season were 0.12 ± 0.02, 0.07 ± 0.01, and 0.00 ± 0.01 kg N_2_O ha^−1^ for the F-RF, F-D-F, and CF plots, respectively (Table 2). In contrast with the abundant rainfall during the early rice season, it was relatively dry during the late rice season. There was one midseason drainage episode during the F-D-F plots in the late rice season, but this only triggered little emission of N_2_O. Relative to the F-D-F plots, the period without a water layer lasted longer in the F-RF plots; consequently, the drying and wetting cycles occurred more frequently in response to the rainfall. This induced greater N_2_O emissions, and the emission peak appeared after N fertilizer topdressing. The cumulative N_2_O fluxes during the late rice season were 0.20 ± 0.03, 0.04 ± 0.02, and −0.01 ± 0.00 kg N_2_O ha^−1^ for the F-RF, F-D-F, and CF plots, respectively (Table 2). Over the annual cycle, the total N_2_O emissions were 0.62 ± 0.09, 0.29 ± 0.05, and −0.01 ± 0.00 kg N_2_O for the F-RF, F-D-F, and CF plots, respectively. Overall, the CF soils acted as negligible N_2_O sinks, and the F-D-F and F-RF soils acted as small N_2_O sources.

### Net annual GWP and GHGI

Significant differences in the cumulative CH_4_ and N_2_O fluxes and SOC sequestration rates were found relative to the three types of water treatment. Trade-offs were found between the CH_4_ and N_2_O emissions and the SOC sequestration. Longer periods of soil flooding brought about increased CH_4_ emissions and decreased N_2_O emissions, and increased amounts of atmospheric CO_2_ were sequestered into soil. However, the proportions of GWP from the CH_4_ and N_2_O emissions and SOC sequestration were quite different. As regards the F-RF soils, the GWP from the CH_4_, N_2_O, and SOC sequestration were 3,816, 184, and −2,354 kg CO_2_-eqv ha^−1^ yr^−1^, respectively. As regards the F-D-F soils, the GWP from the CH_4_, N_2_O, and SOC sequestration were 12,782, 87, and −3,974 kg CO_2_-eqv ha^−1^ yr^−1^, respectively. As regards the CF soils, the GWP from the CH_4_, N_2_O, and SOC sequestration were 27,362, −2, and −4,737 kg CO_2_-eqv ha^−1^ yr^−1^, respectively. On average, the GWP from the CH_4_, N_2_O, and SOC sequestration of the double rice-cropping systems were 14,654, 90, and −3,731 kg CO_2_-eqv ha^−1^ yr^−1^, respectively. This indicates that CH_4_ was a major GWP contributor and was regulated by field water regimes. The GWP decreased with a reduction in the flooding time. Shortening the flooding period greatly reduced the annual CH_4_ emissions, although it resulted in a slight increase in N_2_O emissions and reduced SOC sequestration. Therefore, relative to the CF plots, the net annual GWP was 60% smaller for the F-D-F plots and 93% smaller for the F-RF plots (Table 3). There was no significant difference in the annual rice grain yields between the F-D-F and CF plots. The rice grain yields decreased significantly (by 13%) in the F-RF plots in comparison with the F-D-F plots. Relative to the CF water management, the F-D-F reduced the annual GHGI by 58% and the F-RF reduced annual GHGI by 91% (Table 3).

## Discussion

### Carbon sequestration in double rice-cropping systems

Organic carbon in rice straw and grains would turn into CO_2_ again, and will not be existing as organic material for a long time. Therefore, the CO_2_ balance between the atmosphere and the rice-cropping systems was determined based on the changes in the SOC stock. Consistent with previous reports claiming that rice cultivation accelerated SOC sequestration^11,16–18^, all the plots in this study showed significant increases in the SOC content (Table 1). The SOCSR in the 0–20 cm soil ranged from 501–741 kg C ha^−1^ yr^−1^, which falls within the SOC sequestration rate of 0.13–2.20 t C ha^−1^ yr^−1^ estimated by Pan *et al*.^16^. This is comparable with the range of 0.48–0.70 t C ha^−1^ yr^−1^ reported by Li *et al*.^13^ and the range of 0.38–0.74 t C ha^−1^ yr^−1^ reported by Liu *et al*.^19^, but lower than the range of 0.96–1.11 t ha^−1^ yr^−1^ reported by Shang *et al*. under chemical fertilization in the same study region^11^. The SOCSR in the 20–40 cm soil has rarely been evaluated in previous studies. The SOCSR in the 20–40 cm soil in this study ranged from 141–551 kg C ha^−1^ yr^−1^, which indicated that carbon sequestration in this layer should not be ignored. Several reasons can be attributed to the differences in the SOC sequestration rate between the three treatments. Firstly, biomass production was larger in the F-D-F and CF plots, which resulted in increased amounts of stubble and root residue retained in the soils. Secondly, longer periods of waterlogging slowed the decomposition of SOC. Thirdly, waterlogging accelerated carbon migration deeper into the soil possibly by processes of diffusion and leaching, resulting in substantial SOC sequestration in the 20–40 cm soil layer in the CF and F-D-F treatment.

### CH_4_ emissions from double rice-cropping systems

The CH_4_ emissions were found to be regulated by field water conditions (Fig. 1), which was consistent with previous reports^5,6,20,21^. In the present study, the annual CH_4_ emissions varied between 112 and 805 kg CH_4_ ha^−1^ yr^−1^ (Table 3), which was within the ranges identified by Huang *et al*.^22^ and Li *et al*.^13^. Over the annual cycle, the F-RF, F-D-F, and CF soils were flooded for 121, 175, and 365 days, respectively. It is generally believed that flooding increases the CH_4_ emissions from rice paddies. In the present study, CH_4_ emissions increased significantly with flooding days (r = 0.981, P < 0.01). Continuous waterlogging occurred primarily during the early rice season in all the plots, but the difference in the seasonal CH_4_ emissions from the F-RF, F-D-F, and CF plots was significant (Fig. 1a and Table 2). Similar results have been reported by Cai^23^. This finding can be associated probably with the history of the soil water conditions that introduced a cumulative effect on the physicochemical properties and microorganisms of the soil^24,25^. During the late rice season, the CH_4_ emissions in the F-D-F plots decreased quickly to zero when the midseason drainage applied, and gradually rebounding to a secondary peak after re-flooding (Fig. 1a). This finding is consistent with the observation that the midseason drainage of irrigated rice paddies often causes a drop in the seasonal CH_4_ flux^20,21,23^. Despite the fallow season lasting for up to six months, the cumulative CH_4_ emissions during the fallow season were small, even under continuous flooding conditions (Fig. 1a and Table 2). This can probably be ascribed to low temperatures and the absence of rice plants that accelerate the production of CH_4_ through root exudate and the release of CH_4_ through aerenchyma. The cumulative CH_4_ emissions during the late rice season contributed up to 50–64% of the annual CH_4_ emissions. Relative to the early rice season, the CH_4_ emissions were significantly larger in the late rice season under the F-D-F and CF treatments. Air temperature during late rice season was relatively high, and the late rice season was 16 days longer than the early rice season. Because of the long period of non-flooding in the F-RF plots during the late rice season, the CH_4_ emissions were comparable with those during the early rice season.

### N_2_O emissions from double rice-cropping systems

It is well documented that the N_2_O emission during the rice growing seasons depends significantly on the water regimes^9,11,21,26–29^. Consistent with previous studies, the N_2_O emissions in the present study were negligible when the paddies were flooded during the rice-growing season, and most N_2_O emissions occurred when the water layer disappeared during the fallow season, even though no N fertilizer was applied. Over the entire annual cycle, the CF soils acted as extremely small sinks of N_2_O. During the rice growing seasons, no N_2_O emission peaks appeared after N fertilization, and a similar scenario was found in some other reports^11,30,31^. This is probably because the strictly anaerobic conditions in the flooded paddies are suitable for denitrification, and the major product of denitrification is N_2_^32^. Furthermore, no N_2_O emission peaks appeared during the period of midseason drainage. Although the CH_4_ emissions decreased under the midseason drainage, N_2_O emissions did not arise, which was inconsistent with some previous studies^21,23^. It has been reported that significant CH_4_ emission occurred at soil redox potentials lower than approximately −100 mV, whereas emissions of N_2_O were insignificant when soil redox potentials below +200 mV^33^. In the present study, the soil redox potential during the midseason drainage was probably within the range of −100 and +200 mV, which would be low enough to prevent CH_4_ production and encourage N_2_O reduction to N_2_^33^.

### Net GWP and GHGI of double rice-cropping production

Although GHG emissions from rice paddies have been well documented in the past decades, few estimations of net GWP have been made for Chinese double rice-cropping systems under different long-term water management strategies. Significant positive relationships were found between the annual CH_4_ emissions and the SOCSR in the 0–20 cm (r = 0.810, P < 0.01) and 0–40 cm soils (r = 0.938, P < 0.01), suggesting that the SOC sequestration could stimulate CH_4_ emissions. Significant negative relationship was found between the annual CH_4_ emissions and annual N_2_O emissions (r = −0.924, P < 0.01). However, the net GWP was dominated by CH_4_ emissions, consistent with the findings of previous studies^11,13,14^. Compared with the CF water management, which enhanced the sequestration of atmospheric CO_2_ into the soil, the F-D-F and F-RF water management strategies led to a decrease in the annual CH_4_ emissions of 53% and 86%, respectively, which resulted in a decline in the net annual GWP by 60% and 93%, respectively. Therefore, considerable potential exists for the mitigation of GWP by the adoption of effective water management strategies. The net GWP and GHGI in the present study are lower than the report of Shang *et al*.^11^, mainly because of the lower emissions of CH_4_ and N_2_O. Relative to the CF water management, the F-D-F produced comparable grain yields, but decreased the annual GHGI by 58%; the F-RF water management decreased grain yield by 13%, but the annual GHGI decreased by 91%. This suggests that by adopting appropriate water management strategies, it would be possible to achieve the dual goals of maintaining productivity while minimizing the negative climatic effects of rice cultivation.

In the present study, CH_4_ and N_2_O emissions were investigated from 2014 to 2015, which represented the final situations under different water management strategies throughout 16 years (1998–2014), while the SOCSR were calculated as the average value of 16 years (1998–2014). Since the SOCSR would decrease year by year as the SOC content going closer to the SOC saturation, the usage of SOCSR from 1998–2014 may underestimate the GWP and GHGI.

### Water management strategies in double rice-cropping systems

In the present study, grain yield was comparable between the F-D-F and CF treatments, whereas it was significantly lower (13%) for the F-RF plots (Table 3). This suggests that the F-D-F treatment was suitable for achieving the dual goals of sustaining productivity and minimizing the negative climatic effects in double rice systems. The F-RF treatment was best in mitigation climatic effects, but the yield penalty should be a concern. This finding has implications with respect to water resources. Water management strategies before the rice season have cumulative effects on the physicochemical properties and microorganisms of soils and, consequently, on the GHG emissions. In addition, the soil water conditions during the fallow season are important factors in the mitigation of CH_4_ emissions. Although drainage results in a trade-off between CH_4_ and N_2_O emissions and SOC sequestration, midseason drainage and fallow season drainage should be implemented to greatly reduce CH_4_ emissions during the rice and non-rice seasons and, thereby, to minimize the GWP of the double rice-cropping production. In addition, keeping the fields flooded after the application of nitrogen fertilizers is useful in the mitigation of N_2_O emissions. In an instance of drought and to avoid nutrient runoff, farmers should not drain water needlessly. Based on the results of grain yield, GWP, and GHGI, a water management strategy similar to the F-D-F treatment should be used to maintain grain yields and, simultaneously, mitigate the climatic impacts of double rice-cropping production.

## Conclusions

This study provides a complete assessment of the effects on GWP and GHGI of typical long-term water management regimes in Chinese double rice-cropping systems. The various long-term water management treatments were found to have significant effects on the SOCSR and emissions of the CH_4_ and N_2_O. Longer periods of soil flooding led to higher SOCSR and CH_4_ emissions, whereas the N_2_O emissions were reduced. The net GWP was dominated by CH_4_ emissions. Relative to the CF water management, the F-D-F and F-RF showed remarkable reductions in the net annual GWP of 60% and 93%, respectively. There was no significant difference in the annual rice grain yields between the F-D-F and CF plots. The rice grain yield decreased significantly (by 13%) in the F-RF plots in comparison with that in the F-D-F plots. Relative to the CF water management, F-D-F and F-RF showed remarkable decreases in the annual GHGI of 58% and 91%, respectively. To simultaneously achieve high grain yields and low GHGI, agricultural management strategies that adopt midseason drainage and non-rice season drainage are recommended for double rice-cropping systems in China.

## Materials and Methods

### Experiment site

The field experiment was conducted in an area that is part of a long-term paddy water management experiment at Taoyuan Station of Agro-ecology Research. It is located in a typical area for double rice cropping in southern China (28°55′N, 111°30′E; altitude: 92.2–125.3 m). The region is characterized by a subtropical humid monsoon climate, with annual averages of air temperature, precipitation, sunshine, and frost-free period of 16.51 °C, 1,448 mm, 1,513 h, and 283 days, respectively. The paddy soil is classified as Stagnic Anthrosols^34^ developed from Quaternary red clay.

### Field experiment

The long-term paddy water management experimental grid has been established since 1998. The investigated water management strategies were: (i) continuous year-round flooding with a 2–10 cm water layer (CF); (ii) flooding during the rice season, except for drainage at midseason and harvest time (F-D-F); and (iii) flooding for transplanting and tillering, with no further irrigation (F-RF). There are three replicates for each treatment and the size of each field plot is 6 m × 6 m. The specific details of the field water conditions are shown in Fig. 1c.

The cropping system is fallow–rice–rice. The fertilizers were urea for nitrogen (N), superphosphate for phosphorus (P), and potassium chloride for potassium (K), which were applied at rates of 182 kg N ha^−1^ yr^−1^, 39.3 kg P ha^−1^ yr^−1^, and 198 kg K ha^−1^ yr^−1^, respectively. Urea was applied with two splits for the early rice season, namely, 50% as basal fertilizer and 50% as tillering fertilizer, and with three splits for the late rice season, namely, 50% as basal fertilizer, 33.3% as tillering fertilizer, and 16.7% as panicle fertilizer. The P and K fertilizers were applied as basal fertilizers. The basal fertilizer, applied one day before rice transplanting, was incorporated well into the soil by plowing to 10–20 cm depth, and the topdressing application was surface broadcast. In addition, the use of pesticides and herbicides followed local practices. Local rice (*Oryza sativa* L.) cultivars, *Zhongzao* 39, and *Fengyuanyou* 227 were used for the early and the late rice seasons, respectively. As regards the early crop, rice seedlings were transplanted on April 22 at a hill density of 20 cm × 20 cm and harvested on July 8. As regards the late crop, rice was transplanted on July 15 at a hill density of 20 cm × 25 cm and harvested on October 16. After the harvest, the rice straw was removed, leaving stubble approximately 10 cm long in the plots.

### Measurements of CH_4_ and N_2_O fluxes

The measurements of the CH_4_ and N_2_O emissions were recorded from November 2014 to October 2015, throughout an annual fallow–rice–rice cycle. The CH_4_ and N_2_O fluxes were measured by using a static closed-chamber method. A rectangular sampling chamber (60 cm wide × 60 cm long × 100 cm high) was constructed of sandwich foam plate to minimize air temperature changes inside the chamber during the sampling. A single 12 V fan was installed inside the chamber for the mixing of the gas. In each plot, a chamber-base collar (60 cm wide × 60 cm long) made of polyvinyl chloride plate was fixed into the soil at a depth of 15 cm and was retained in place, except during tillage before transplanting. Gas samples were taken between 09:20–10:40 local time. To calculate the gas change rate, at each plot, gas samples of approximately 30 mL of gas were injected into pre-evacuated vials using a syringe at 0, 15, 30, 45, and 60 min. The air temperature inside the chamber was monitored during the gas collection. Generally, samples were collected every 6 days during the rice season (except for a 3–4 days interval after fertilization) and every 10 days during the fallow season. The concentrations of CH_4_ and N_2_O were analyzed by using a gas chromatograph (Agilent 7890A, Agilent Technologies, USA). Gas fluxes were calculated according to the linear change in gas concentration with sampling time, chamber headspace height, air pressure, and air temperature within the chambers^30,35^. The cumulative gas emissions was sequentially accumulated from the emissions between every two sequential intervals of the gas fluxes^21,30^.

### SOC sequestration estimates

The soil samples were collected at depths of 0–20 and 20–40 cm before the early rice transplanting in 1998 and 2014. Twelve soil cores were collected from each plot using augers (3 cm diameter) and mixed to provide a single sample. Visible plant detritus and any fragments were removed after air-drying at room temperature. Each soil sample was ground to pass through a 0.15 mm sieve for the analysis of SOC, which was determined by potassium dichromate oxidation titration. Four soil cores from each plot and from each soil depth (0–20 and 20–40 cm) were collected by using stainless steel cylinders to measure the soil bulk density (γ). Because soil bulk density has high spatiotemporal variability, we calculated the SOC stock on an “identical soil mass” basis rather than on the fixed soil thickness. The SOC stocks in the 0–20 cm and 20–40 cm soil layers were estimated according to the procedure provided by Nishimura *et al*.^36^. The SOC sequestering rate (SOCSR) was estimated with the following equation:

1 $${\rm{SOCSR}}=({{\rm{CS}}}_{{\rm{t}}2}-{{\rm{CS}}}_{{\rm{t}}1})/({{\rm{t}}}_{2}-{{\rm{t}}}_{1})$$

where CS_t1_ is C stock in t_1_ (1998), and CS_t2_ is C stock in t_2_ (2014).

### Net GWP and GHGI estimates

Global warming potential with a 100-year time horizon was converted into CO_2_-equivalent emissions by multiplying the cumulative emissions of CH_4_ and N_2_O by 34 and 298, respectively^37^.

2 $${\rm{GWP}}=34\times {{\rm{CH}}}_{4}+298\times {{\rm{N}}}_{2}{\rm{O}}-{\rm{SOCSR}}\times 44/12\,({\rm{kg}}\,{{\rm{CO}}}_{2}-{\rm{eqv}}\,{{\rm{ha}}}^{-1}\,{{\rm{yr}}}^{-1})$$

Subsequently, the GHGI was calculated by dividing GWP by the rice grain yield^14,15,38^.

3 $${\rm{GHGI}}={\rm{GWP}}/{\rm{grain}}\,\mathrm{yield}\,(\mathrm{kg}\,{{\rm{CO}}}_{2}-{\rm{eqv}}\,{{\rm{kg}}}^{-1}\,\mathrm{grain})$$

### Other data measurements

The floodwater depth was measured by using a steel ruler. All the rice in each plot was harvested manually. Grain samples were oven-dried at 70 °C and weighed to calculate the crop grain yields assuming water content of 14%.

### Statistical analyses

All statistical analyses were performed with SPSS 17.0 (SPSS, Inc., USA) using ANOVA, followed by the least significant difference (LSD) test, in which P < 0.05 was considered statistically significant. The relationships between seasonal cumulative CH_4_ and N_2_O fluxes and the SOC sequestration rate and flooding days were evaluated by using linear regression.
